# Calcium release channel deficiency syndrome in patients diagnosed with idiopathic ventricular fibrillation and decedents classified as sudden unexplained death in the young

**DOI:** 10.1093/europace/euaf303

**Published:** 2026-03-05

**Authors:** Lucilla Giammarino, Raquel Neves, David J Tester, Sahej Bains, Vanessa Karlinski Vizentin, J Martijn Bos, John R Giudicessi, Michael J Ackerman

**Affiliations:** Departments of Cardiovascular Medicine, Pediatric and Adolescent Medicine, and Molecular Pharmacology & Experimental Therapeutics, Divisions of Heart Rhythm Services and Pediatric Cardiology, Windland Smith Rice Genetic Heart Rhythm Clinic and the Windland Smith Rice Sudden Death Genomics Laboratory, Mayo Clinic, Guggenheim 501, Rochester, MN 55905, USA; Departments of Cardiovascular Medicine, Pediatric and Adolescent Medicine, and Molecular Pharmacology & Experimental Therapeutics, Divisions of Heart Rhythm Services and Pediatric Cardiology, Windland Smith Rice Genetic Heart Rhythm Clinic and the Windland Smith Rice Sudden Death Genomics Laboratory, Mayo Clinic, Guggenheim 501, Rochester, MN 55905, USA; Departments of Cardiovascular Medicine, Pediatric and Adolescent Medicine, and Molecular Pharmacology & Experimental Therapeutics, Divisions of Heart Rhythm Services and Pediatric Cardiology, Windland Smith Rice Genetic Heart Rhythm Clinic and the Windland Smith Rice Sudden Death Genomics Laboratory, Mayo Clinic, Guggenheim 501, Rochester, MN 55905, USA; Departments of Cardiovascular Medicine, Pediatric and Adolescent Medicine, and Molecular Pharmacology & Experimental Therapeutics, Divisions of Heart Rhythm Services and Pediatric Cardiology, Windland Smith Rice Genetic Heart Rhythm Clinic and the Windland Smith Rice Sudden Death Genomics Laboratory, Mayo Clinic, Guggenheim 501, Rochester, MN 55905, USA; Departments of Cardiovascular Medicine, Pediatric and Adolescent Medicine, and Molecular Pharmacology & Experimental Therapeutics, Divisions of Heart Rhythm Services and Pediatric Cardiology, Windland Smith Rice Genetic Heart Rhythm Clinic and the Windland Smith Rice Sudden Death Genomics Laboratory, Mayo Clinic, Guggenheim 501, Rochester, MN 55905, USA; Departments of Cardiovascular Medicine, Pediatric and Adolescent Medicine, and Molecular Pharmacology & Experimental Therapeutics, Divisions of Heart Rhythm Services and Pediatric Cardiology, Windland Smith Rice Genetic Heart Rhythm Clinic and the Windland Smith Rice Sudden Death Genomics Laboratory, Mayo Clinic, Guggenheim 501, Rochester, MN 55905, USA; Departments of Cardiovascular Medicine, Pediatric and Adolescent Medicine, and Molecular Pharmacology & Experimental Therapeutics, Divisions of Heart Rhythm Services and Pediatric Cardiology, Windland Smith Rice Genetic Heart Rhythm Clinic and the Windland Smith Rice Sudden Death Genomics Laboratory, Mayo Clinic, Guggenheim 501, Rochester, MN 55905, USA; Departments of Cardiovascular Medicine, Pediatric and Adolescent Medicine, and Molecular Pharmacology & Experimental Therapeutics, Divisions of Heart Rhythm Services and Pediatric Cardiology, Windland Smith Rice Genetic Heart Rhythm Clinic and the Windland Smith Rice Sudden Death Genomics Laboratory, Mayo Clinic, Guggenheim 501, Rochester, MN 55905, USA

**Keywords:** Arrhythmias, Calcium release channel deficiency syndrome, Genetics, *RYR2*, Sudden cardiac arrest, Syncope

## Abstract

**Aims:**

Calcium release channel deficiency syndrome (CRCDS) results from loss-of-function (LOF) variants in the RYR2-encoded type 2 ryanodine receptor (RyR2), predisposing patients to sudden cardiac arrest/death (SCA/SCD) without abnormalities on a stress electrocardiogram (ECG). Undetected CRCDS may underlie idiopathic ventricular fibrillation (IVF) and sudden unexplained death in the young (SUDY). We aimed to determine the prevalence of potential CRCDS-causative *RYR2* variants in IVF and SUDY.

**Methods and results:**

We reviewed clinical evaluation and *RYR2* genetic analysis of 169 IVF patients and 279 SUDY victims. Only ultra-rare (<0.005% in gnomAD) nonsynonymous *RYR2* variants were considered potentially pathogenic. Among IVF patients, 6/169 (3%) overall—and 6/67 (9%) with exertion-related SCA—harboured an *RYR2* variant and represent potential CRCDS cases. All exhibited normal resting and stress ECGs. Genetic analysis revealed six distinct *RYR2* variants, two previously characterized as LOF. In SUDY, 31/279 victims (11%) had a *RYR2* variant (30 unique variants), predominantly observed in exertion-related SCD 20/83 (24%) vs. rest-related 11/196 (6%). Of the 14 SUDY victims with functionally characterized *RYR2* variants, five (2% of total cohort) had a LOF variant; among the 56 exertion-related SUDY cases, four (7%) had a LOF variant.

**Conclusion:**

CRCDS may account for 3% of IVF overall and 9% of exertion-related SCA in IVF. Ultra-rare *RYR2* variants may underlie up to 11% of SUDY, with 65% of *RYR2*-positive cases occurring during exertion. LOF-*RYR2* variants may contribute to ≥7% of exercise-associated SUDY. Accurate identification of the underlying ryanodinopathy is essential for clinical management of affected patients.

## Graphical Abstract

**Graphical Abstract:**
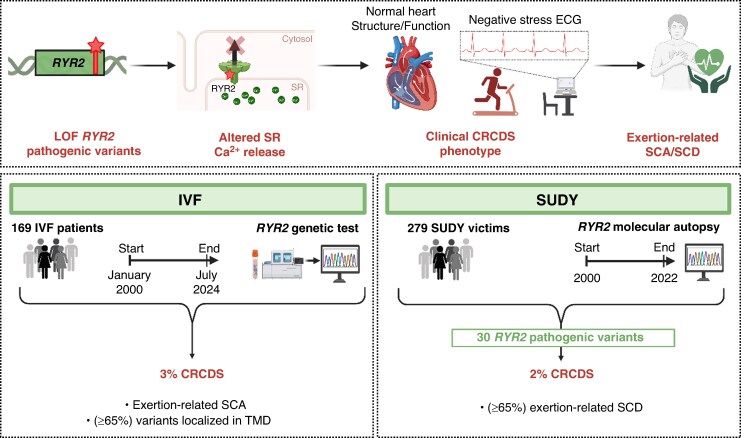
Prevalence and clinical presentation of calcium release channel deficiency syndrome (CRCDS) in idiopathic ventricular fibrillation (IVF) and sudden unexplained death in the young (SUDY). Loss-of-function (LOF) pathogenic variants in the *RYR2* gene alter physiological sarcoplasmic reticulum (SR) calcium (Ca²⁺) release, causing CRCDS. Despite preserved cardiac structure/function and negative stress ECGs, CRCDS predisposes individuals to exertion-related sudden cardiac arrest (SCA) or sudden cardiac death (SCD). In this study, 169 IVF patients underwent *RYR2* genetic testing, revealing a 3% prevalence of potential CRCDS cases, of whom all were associated with exertion-related SCA. Molecular autopsy of 279 SUDY victims identified 30 *RYR2* pathogenic variants in 31 cases overall (11%), with at least 2% of cases possessing CRCDS-causing variants and predominantly associated with exertion-related SCD. *Abbreviations*: Ca²⁺, calcium ions; CRCDS, calcium release channel deficiency syndrome; ECG, electrocardiogram; IVF, idiopathic ventricular fibrillation; LOF, loss-of-function; *RYR2*, ryanodine receptor 2 (gene); RyR2, ryanodine receptor 2 (protein), SCA, sudden cardiac arrest; SCD, sudden cardiac death; SR, sarcoplasmic reticulum; SUDY, sudden unexplained death in the young; TMD, transmembrane domain.

What’s new?

- We assessed the prevalence of CRCDS in two large unrelated cohorts: 169 IVF patients and 279 SUDY victims.
- We described the clinical phenotype of CRCDS patients presenting as IVF, showing negative stress test ECG despite the occurrence of exertion-related SCA.
- We demonstrated that sudden VF onset in CRCDS cases contrasts with the typical arrhythmia progression observed in patients with the more common *RYR2-*mediated type 1 catecholaminergic polymorphic ventricular tachycardia.

## Introduction

Calcium release channel deficiency syndrome (CRCDS), also referred to as calcium release deficiency syndrome (CRDS), is a recently described arrhythmia syndrome caused by loss-of-function (LOF) pathogenic variants in the *RYR2*-encoded type 2 ryanodine receptor (RyR2)/calcium release channels (CRCs).^1–4^ These CRCs localize to the sarcoplasmic reticulum (SR) and are critical for excitation–contraction coupling. During a cardiac action potential (AP), the opening of voltage-gated L-type calcium channels triggers conformational changes and consequential activation of the CRCs due to sarcolemmal depolarization. This process leads to a substantial release of calcium ions (Ca^2+^) from the SR to the cytoplasm—a mechanism known as Ca^2+^-induced Ca^2+^ release (CICR)—that amplifies the initial Ca^2+^ signal (I_CaL_) crucial for cardiac contraction.^5^

Alterations in RyR2-mediated SR Ca^2+^ release, occurring during either systole or diastole, can result in contractile dysfunction and electrical instability.^6^ These abnormalities in intracellular Ca^2+^ handling contribute to the development of potentially life-threatening ventricular arrhythmias (VAs).^7^ Among RyR2-channelopathies, gain-of-function (GOF) pathogenic variants are the cause of type 1 catecholaminergic polymorphic ventricular tachycardia (CPVT1), while LOF variants are responsible for CRCDS. Recent studies have shown that CRCDS-causative RyR2 variants impair Ca^2+^ transient and promote progressive SR Ca^2+^ overload.^2,8–10^

As in CPVT, patients with CRCDS present with structurally normal hearts, however CRCDS patients experience sudden cardiac arrest (SCA) in the absence of electrical abnormalities during exercise stress testing.^11^ In these patients, the first clinical manifestation is often SCA or sudden cardiac death (SCD).^12^ As a result, a subset of patients with idiopathic ventricular fibrillation (IVF) may represent undiagnosed CRCDS. However, the true prevalence and clinical spectrum of CRCDS remain poorly defined, due to the limited functional data on CRCDS-causative RyR2 variants and the lack of a validated clinical diagnostic approach.^11,13^

In addition, pathogenic RyR2 variants account for a significant proportion of autopsy-negative sudden unexplained death in the young (SUDY) cases,^14,15^ commonly defined as SCD occurring before 40 years of age.^16^ Accurate identification of the underlying type of cardiac ryanodinopathy is critical for clinical diagnosis and management of surviving family members. Molecular autopsy has proven highly valuable in this setting: post-mortem genetic testing in SUDY victims demonstrates a high diagnostic yield for inherited cardiac diseases, enabling targeted risk reduction, family counselling, and preventive treatment.^17^ Similarly, in athletes who died suddenly and underwent molecular autopsy, a diagnostic yield of approximately 17% was reported.^18^ However, although access continues to improve, the availability of genetic testing remains heterogeneous and not globally accessible, often requiring referral to specialized centres, although access continues to improve.^19^ Despite these advances, the relative contribution of functionally characterized *RYR2*-pathogenic variants associated with CRCDS vs. CPVT1 identified in SUDY victims remains unclear.

As such, we conducted a retrospective analysis of a large cohort of patients with IVF to assess the contribution of RyR2-mediated CRCDS to the IVF phenotype as well as the contribution of functionally characterized CRCDS- and CPVT1-associated RyR2 variants in a large cohort of unrelated SUDY cases.

## Methods

The research reported in this article followed the Declaration of Helsinki (as revised in 2013) and adhered to the CARE case report guidelines.

### Idiopathic ventricular fibrillation cohort

In this Mayo Clinic Institutional Review Board-approved retrospective study, we reviewed the available electronic medical records of 169 patients evaluated and treated at Mayo Clinic’s Windland Smith Rice Genetic Heart Rhythm Clinic between January 2000 and July 2024, who received a default diagnosis of IVF following a comprehensive cardiac evaluation that included: ECG, Holter, cardiac MRI, and electrophysiology study when indicated in association with genetic testing for channelopathy and cardiomyopathy-causative genes. Genetic variants were annotated using the single-letter amino acid nomenclature. A potential CRCDS diagnosis was considered if they met both of the following criteria: (i) no evidence of bidirectional progressive ventricular ectopy with increasing workload during a standard exercise stress ECG test, and (ii) presence of an ultra-rare—minor allele frequency < 5 × 10^−5^ in the genome aggregation database (gnomAD)—nonsynonymous variant within *RYR2;* synonymous single-nucleotide variants were excluded.

### *RYR2* molecular autopsy in SUDY victims

A previously genotyped cohort of 279 SUDY victims was reviewed.^20–27^ Research-based post-mortem *RYR2* genetic testing had been performed using a combination of first- and next-generation sequencing platforms. Only ultra-rare (minor allele frequency < 5 × 10^−5^ in gnomAD) nonsynonymous variants within *RYR2* were considered. For this study, SUDY is defined as SCD occurring in individuals aged 1–35 years, thereby excluding sudden infant death syndrome cases (age 0−1). A review of the literature identified RyR2 variants that have been characterized functionally as either LOF or GOF.

## Results

### Idiopathic ventricular fibrillation cohort

The IVF cohort consisted of 169 patients [68 female (40%), 101 males (60%), mean age of 31 ± 14 years at SCA]. The demographics are summarized in *Table 1*. A positive family history of SCD was reported in 31 patients (18%), and most cardiac events (*n* = 102; 60%) occurred at rest. All patients had a structurally normal heart, with a mean left ventricle ejection fraction (LVEF) of 60 ± 5% and normal ECG findings, with a mean QTc of 421 ± 30 ms. The cardiac event occurring in the setting of exertion in 67 patients (40%). Recurrent cardiac events occurred in 77 patients (46%).

**Table 1:** Demographics of the total IVF cohort and the potential CRCDS cases

	IVF cohort (*n* = 169)	CRCDS (*n* = 6)
Demographics		
Female, *n* (%)	68 (40)	3 (50)
Male, *n* (%)	101 (60)	3 (50)
Average age at SCA, y	31 ± 14	23 ± 2
Age range, y	1–73	8–40
Recurrent cardiac events, *n* (%)	77 (46)	1 (17)
History of syncope/seizure, *n* (%)	40 (24)	2 (33)
Family history of SCD, *n* (%)	31 (18)	1 (17)
Activity at cardiac event, *n* (%)		
Rest	102 (60)	0 (0)
Exertion	67 (40)	6 (100)
Electrocardiographic and echocardiographic findings		
Average QTc, ms	421 ± 30	434 ± 18
Average LVEF, %	60 ± 5	60 ± 2
Additional work-up, *n* (%)		
Coronary angiography (invasive or CT)	79 (47)	1 (17)
Cardiac MRI	113 (67)	3 (50)
EP study	75 (44)	2 (33)
Device therapy, *n* (%)		
ICD implanted	164 (97)	6 (100)
Appropriate ICD shocks	77 (47)	3 (50)

Mean ± Std. Deviation.

CRCDS, calcium release channel deficiency syndrome; CT, computed tomography; EP, electrophysiological; ICD, implantable cardioverter defibrillator; IVF, idiopathic ventricular fibrillation; LVEF, left ventricular ejection fraction; MRI, magnetic resonance imaging; QTc, corrected QT interval; SCA, sudden cardiac arrest; SCD, sudden cardiac death.

**Figure 1:**
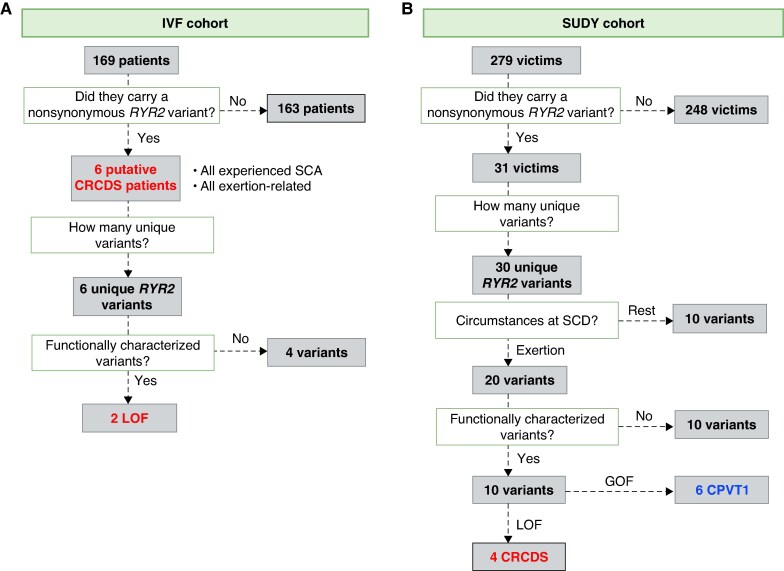
Flow charts of patient selection and ultra-rare nonsynonymous *RYR2* variants in the two cohorts. (*A*) IVF cohort: Among 169 IVF patients, six were identified as potential CRCDS cases, based on stress-test ECG and genetic findings. All experienced exertion-related SCA and carried six unique *RYR2* variants, two of which were previously characterized as LOF. (*B*) SUDY cohort: Among 279 SUDY victims, 31 carried a *RYR2* variant, corresponding to 30 unique variants. Of these, 20 variants were associated with exertion-related SCD. Functional data were available for 10 variants: 6 were GOF (CPVT1-associated) and 4 were LOF (CRCDS-associated).

Genetic analysis revealed six distinct RyR2 variants (p.E243K, p.R4608Q, p.R4790Q, p.Y4813C, p.G4828R, p.G4935R), all initially classified as variants of uncertain significance (VUS) (*Figure 1*) in 6/169 (3%, 95% CI: 1.3–7.6%) patients with IVF. Of these, two (p.R4608Q and p.Y4813C) were *de novo* and two (p.R4608Q and p.G4935R) have been previously functionally characterized as LOF variants and associated with CRCDS.^21,22^

**Figure 2:**
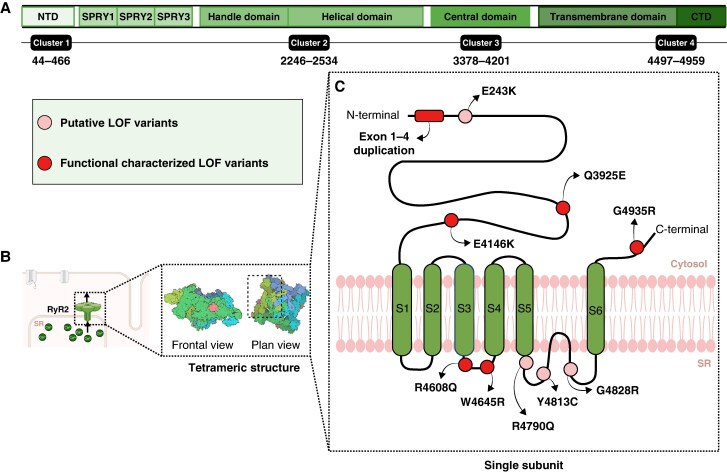
Schematic representation of the *RYR2* gene and RyR2 protein depicting all CRCDS-associated variants identified in our SUDY and IVF cohorts. (*A*) Linear *RYR2* map indicating domains and variant clusters. (*B*) Tetrameric RyR2 structure representing the phosphorylated human RyR2 (closed state; PDB ID: 7U9Q*^28^*). (*C*) Single RyR2 subunit topology showing the localization of all CRCDS-associated *RYR2* variants identified in this study in both cohorts.

**Figure 3:**
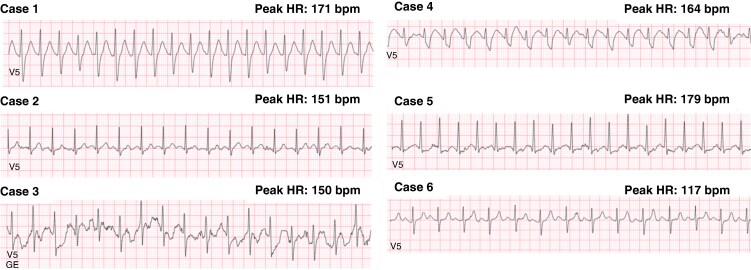
Surface stress-test ECG traces obtained at peak exercise in the six potential CRCDS within our IVF cohort. Representative 12-lead surface stress ECGs are shown for each patient, similar recordings taken from lead V5 at the peak of exercise. Peak HR reached per patient is specified.

Based on absence of bidirectional progressive ventricular ectopy with increasing workload during a standard exercise stress ECG test and the presence of an ultra-rare nonsynonymous variant within *RYR2,* 6/169 (3%, 95% CI: 1.3–7.6%) patients with IVF were considered to be putative CRCDS, including three females (mean age 36 ± 5 years) and three males (mean age 10 ± 4 years) (*Table 1*, *Figure 2A*). Interestingly, all six cases presented SCA in the context of exertional or recreational activities, representing 9% (6/67, 95% CI: 3.3–18.4%) of the patients with exertion-triggered IVF. In four cases, SCA was the first clinical manifestation; in two, syncope preceded SCA. These six individuals exhibited a structurally normal heart with preserved LVEF. Despite previous SCA, their surface 12-lead resting and exercise ECGs were unremarkable. All six putative CRCDS patients received treatment with an implantable cardioverter defibrillator (ICD), and their pharmacological management included nadolol and/or flecainide. Surface stress test ECG in each putative CRCDS is shown in *Figure 3*. Additional details about the six cases are provided in *Table 2* and discussed separately.

**Table 2:** Clinical characteristics of the six individuals with suspected CRCDS

Case	RyR2 variant	RyR2 mutation cluster	Age at sentinel event (y)	Sex	Activity preceding symptoms	Stress ECG results	Echocardiographic findings, LVEF (%)	Most recent therapy prescribed
1	E243K	1	14	Male	Exertional (swimming)	Normal	Normal, 58	Flecainide, LCSD, ICD
2	R4608Q	4^a^	8	Male	Recreational (vigorous play)	Normal	Normal, 62	Nadolol, ICD
3	R4790Q	4^a^	40	Female	Exertional (triathlon)	Single PVC during recovery phase	Normal, 62	Nadolol, ICD
4	Y4813C	4^a^	31	Female	Exertional (post-coital)	Normal	Childhood-onset mild biventricular CMP, 57	Nadolol, ICD
5	G4828R	4^a^	38	Female	Exertional (indoor cycling)	Short-coupled PVC-triggered pVT	Normal, 59	Nadolol, flecainide and ICD
6	G4935R	4	8	Male	Recreational (playing on the playground)	Normal	LV noncompaction, 62	Nadolol, flecainide and ICD

Each case is categorized by *RYR2*-variant and its localization, patient demographics, circumstances preceding SCA, diagnostic findings (ECG stress test and echocardiographic findings), and the most recent prescribed therapy. Mutation clusters were defined as follows: cluster 1 from amino acids 44 to 466, cluster 2 from 2245 to 2534, cluster 3 from 3778 to 4201, cluster 4 from 4497 to 4959.

CMP, cardiomyopathy; LCSD, left cardiac sympathetic denervation; LV, left ventricle; LVEF, left ventricular ejection fraction; pVT, polymorphic ventricular tachycardia; PVC, premature ventricular contraction; SCA, sudden cardiac arrest; TMD, transmembrane domain; VT, ventricular tachycardia.

^a^Indicates *RYR2*-variant localization in the TMD.

### Clinical presentation of patients with putative CRCDS

The first case was a male patient who experienced a swimming-related SCA at 14 years of age. Initial cardiac evaluation revealed a normal ECG (QTc 431 ms) and a structurally normal heart (LVEF 58%). Stress test ECG was performed while on flecainide; 87% of the predicted heart rate (HR) was achieved without occurrence of premature ventricular contraction (PVC). He was managed with an ICD, left cardiac sympathetic denervation (LCSD) and nadolol, which was sub-sequentially changed to flecainide due to nadolol side effects. He later had recurrent appropriate ventricular tachycardia/ventricular fibrillation (VT/VF)-terminating ICD therapies, in the setting on β-blocker noncompliance or emotional stress. Genetic testing revealed a nonsynonymous missense variant p.E243K localized in the N-terminal portion of RyR2 present on the paternal side of the family and that appeared to co-segregate with the VA phenotype. Two additional VUS were detected in RyR2: p.P828S and p.N1503D, located in SPRY2 and SPRY3 domains, respectively. However, neither of these ultra-rare nonsynonymous RyR2 variants appeared to co-segregate with the phenotype in the pedigree (see Supplementary material online, *Figure S1*).

The second case was a male who experienced recreational activity-associated SCA at the age of 8 years during vigorous play with his siblings. During clinical evaluation, he presented with an electrical and structural normal heart (QTc 399 ms; LVEF 62%). His stress test ECG was performed on nadolol, where 72% of the predicted HR was achieved and no electrical abnormalities were observed. He has been treated with nadolol and remains free of cardiac events during a follow-up of 1 year. Genetic testing revealed a *de novo* variant, p.R4608Q, localized in the transmembrane domain (TMD) of the CRC. Previous *in vitro* functional analysis characterized this variant as LOF and associated with CRCDS.^29^

The third case was a female patient who experienced exertional SCA during a triathlon at 40 years of age. Cardiac evaluation demonstrated a normal resting ECG (QTc 448 ms) and echocardiogram (LVEF 62%). Her stress test ECG on nadolol reached 98% of predicted HR and was negative except for a single PVC during the recovery phase. She received an ICD and later had recurrent appropriate VT/VF-terminating ICD shocks. Cardiac electrical evaluation was performed through a classical invasive electrophysiological (EP) study, but no arrhythmia was revealed. She has been treated with nadolol and did not have any cardiac events during a follow-up of 3 years. Genetic testing revealed an RyR2 variant, p.R4790Q, localized in the TMD.

The fourth case was a female patient who presented a post-coital SCA at age 31 years. She had a childhood history of mild biventricular cardiomyopathy with recovered function (LVEF 57%). During a cardiac evaluation ECG showed physiological morphology (QTc 445 ms). Stress test was performed on nadolol, and she achieved 88% of the predicted HR with no PVC occurred. She is being treated pharmacologically with nadolol and has an ICD. During the follow-up of 1 year, while pregnant, she experienced a breakthrough VF event during intercourse, with device interrogation confirming a PVC-triggered VF. Genetic testing revealed a *de novo* RyR2 variant, p.Y4813C, localized in the TMD.

The fifth case is a female presenting with syncope as her sentinel event at age 38 years while jogging. Following her syncopal event, she received an implantable looper recorder (ILR). She had a SCA at age 39 years in the setting of indoor cycling and ILR interrogation revealed PVC-triggered polymorphic VT. Her ECG showed a QTc of 441 ms and her echocardiogram showed a LEVF of 59%. No PVCs were registered during a stress test performed without any medication. During cardiac evaluation, an EP study was performed using the CRCDS protocol (long burst, long pause, short-coupled); however, despite T-wave ballooning, VAs were not induced. She has been treated with nadolol and flecainide. No cardiac events occurred during the follow-up of 2 years. Genetic testing revealed a RyR2 variant, p.G4828R, localized in the TMD.

**Figure 4:**
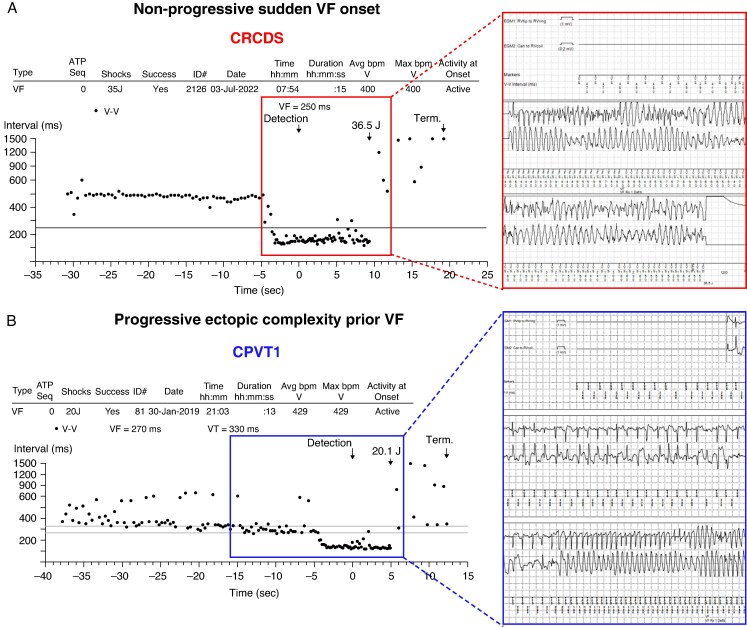
VF onset in a CRCDS patient as compared to a CPVT1 patient. Representative ICD tracings showing in two patients carrying a *RYR2*-pathogenic variant functionally characterized as either LOF in (*A*), or as GOF in (*B*). Arrows indicate detection and initiation of high-voltage defibrillation therapy (expressed in joules). (*A*) CRCDS patient (case 6, IVF cohort) showing sudden VF onset without preceding PVCs or arrhythmic progression detected prior major cardiac event. (*B*) CPVT1 patient (not part of the IVF cohort) demonstrating progressive, increasing ectopic activity and arrhythmic complexity preceding VF and subsequential ICD shock.

The sixth case was a male patient who experienced syncope at age 7 years and 1 year later a SCA while playing on the playground. During cardiac evaluation he presented with a normal ECG (QTc 440 ms) and structurally normal heart (LVEF 62%). His stress test performed on nadolol, where 82% of the predicted HR was achieved, was normal, showing only isolated PVCs. There was no family history of inherited arrhythmia syndromes. He was managed pharmacologically with nadolol and flecainide and received an ICD. He received annual appropriate ICD shocks during a follow-up of 9 years (*Figure 4A*). During recent echocardiographic analysis LV noncompaction was described. Genetic testing revealed a RyR2 variant, p.G4935R, localized in the C-terminal region. Previously, p.G4935R was characterized as LOF and associated with CRCDS.^30^

### Sudden unexplained death in young cohort

**Figure 5:**
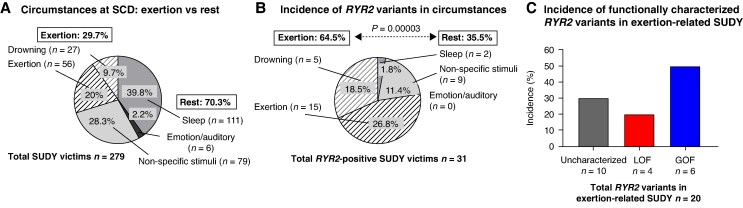
Autonomic contribution in SUDY and *RYR2*-positive SUDY cases. (*A*) Distribution of SCD autonomic triggers (exertion vs. rest) among all SUDY victims. (*B*) Distribution of autonomic triggers (exertion vs. rest) in *RYR2*-positive SUDY victims. Fisher’s exact test. (*C*) Proportion of CRCDS and CPVT1 cases among exertion-related *RYR2*-positive SUDY victims.

The cohort included 279 SUDY victims, consisting of 174 (62%) males and 105 (38%) females (84% white, 174 males, average age = 16 ± 9 years, age range 1–35 years). Most individuals were Caucasian (235 cases, 84%), followed by black (20 cases, 7%) and Asian (9 cases, 3%) individuals; the remaining cases represented other ethnicities, each <3% of the cohort. Mean age at SCD was 16 ± 8 years, with the highest incidence (128 cases, 46%, 95% CI: 40–52%) occurring in individuals between 11 and 19 years of age. SCD occurred during exertion in 83 cases (30%, 95% CI: 24–35%) [exertion or drowning], whereas 196 cases occurred at rest (70%, 95% CI: 65–76%) [sleep or triggered by emotional, auditory or nonspecific stimuli] (*Figure 5A*). The demographics for the 279 SUDY victims are summarized in *Table 3*.

**Table 3:** Demographics and circumstances of death in the SUDY cohort and RYR2-positive SUDY subgroup

	SUDY cohort (*n* = 279)	*RYR2* positive (*n* = 31)
Demographics		
Female, *n* (%)	105 (38)	15 (48)
Male, *n* (%)	174 (62)	16 (52)
Average age at SCD, y	16.1 ± 8.6	15.6 ± 8.2
Personal or family history of cardiac events, *n* (%)	103 (37)	17 (55)
Age range, *n* (%)		
1–10 years	64 (23)	10 (32)
11–19 years	128 (46)	13 (42)
20–35 years	87 (31)	8 (26)
Activity at SCD, *n* (%)		
Exertion	56 (20)	15 (48)
Drowning	27 (10)	5 (16)
Sleep	111 (40)	2 (6)
Nonspecific	79 (28)	9 (29)
Emotion/auditory	6 (2)	0 (0)

SCD, sudden cardiac death.

### Incidence and functional classification of ultra-rare *RYR2* variants in SUDY victims

Overall, 30 unique and ultra-rare nonsynonymous variants in *RYR2* were identified in 31/279 SUDY victims (11%, 95% CI: 8–15%, 16 males, average age, 16 ± 8 years, Supplementary material online, *Table S1*). One individual had two ultra-rare RyR2 variants (p.H240R and p.T4158P), and two variants (p.R420W and p.S2246L) were each observed in two different individuals, resulting in 30 unique variants overall. Segregation analysis was feasible in only 12 SUDY cases due to unavailability of parental DNA, of which 8 variants were inherited and 4 were *de novo* (see Supplementary material online, *Table S1*). The yield was significantly higher (24%, 95% CI: 16–35%, 20/83% vs. 6%, 95% CI: 3–10%, 11/196, *P* = 0.00003, *Figure 5B*) among exertional SUDY cases (27%, 95% CI: 16–41%, 15/56) or drownings (18.5%, 95% CI: 6–385, 5/27) compared to other triggers combined [nonspecific (11.4%, 95% CI: 5–20%, 9/79), sleep (2%, 95% CI: 0–7%, 2/111), emotion/auditory (0%, 95% CI: 0–45%, 0/6)]. So far, 12 of these 30 unique variants have been functionally characterized: 7 as GOF variants having a GOF and 5 with LOF (see Supplementary material online, *Table S1*).

Among the 14 SUDY victims with a functionally characterized RyR2 variant, 9 (3% of the total cohort, 95% CI: 1–6%) had a CPVT1-like GOF variant while 5 (2% of the total cohort, 95% CI: 1–4%) had a CRCDS-like LOF variant. Of the functionally characterized RyR2-LOF variants, 2 were *de novo* and 1 had a recessive inheritance. Among the 20 *RYR2-*positive exertional or drowning related SUDY cases, 10 (50%, 95% CI: 28–72%) had an uncharacterized variant while 6 (30%, 95% CI: 12–54%) had a GOF variant and 4 had a LOF (20%, 95% CI: 6–44%) variant (*Figure 5C*). The average age of SUDY victims with a CRCDS-predisposing LOF variant was 9.2 ± 3.3 years compared to 15.3 ± 11.2 years for those with a CPVT1-susceptibility variant (*P* = 0.26).

## Discussion

CRCDS is a recently described congenital arrhythmia syndrome caused by pathogenic LOF variants in the *RYR2-*encoded RyR2/CRC, leading to SCA and SCD in otherwise healthy individuals. Its clinical presentation is often elusive and typically undetectable through standard cardiac evaluation; moreover, the spectrum of causative pathogenic variants remains incompletely identified. As a result, a definitive CRCDS diagnosis requires identification of pathogenic *RYR2* variants, supported by *in vitro* functional characterization.^13,31^ Notably, not all LOF RyR2-variant positive individuals manifest the same clinical phenotype, highlighting CRCDS’ marked variable expressivity and heterogeneity.^32^ Santiago and Priori described striking intra-familial variable expressivity in individuals heterozygous for the LOF RyR2 pathogenic variant p.D4646A, with phenotype ranging from asymptomatic to SCD.^9,33^ Similarly, in our centre, we observed a comparable pattern in families with a LOF *RYR2* variant, in which the proband experienced a major cardiac event (SCA or SCD) while other variant-positive relatives remained asymptomatic. In addition to major cardiac events, CRCDS patients may present with QT prolongation, bradycardia, or cardiac structural abnormalities (e.g. LV non-compaction and dilated cardiomyopathy).^34–36^

In stark contrast to patients with CPVT1, individuals with CRCDS—despite experiencing life threatening and fatal VAs during exercise—typically exhibit no increase in PVCs during exercise stress test.^9,31,36,37^ In our study, we identified six potential CRCDS cases among IVF patients (3%), all of whom experienced catecholamine-triggered SCA without the pathognomonic CPVT1 hallmark of frequent and progressively complex PVCs during increasing workload and HR. Notably, CRCDS accounted for up to 9% of IVF patients who experienced a cardiac event during physical exertion. These findings underscore the importance of investigating CRCDS in patients with exercise-related SCA; particularly those harbouring a VUS in *RYR2*.

In 2021, Sun and colleagues developed a ventricular stimulation protocol—referred to as LBLPS (long-burst, long-pause, short-coupled extra-stimulus) as a potential diagnostic test for CRCDS.^9^ However, it may have only modest diagnostic sensitivity. This protocol was designed to mimic the arrhythmogenic pattern captured by the ICDs prior to delivery of a successful VF-terminating shock in affected individuals.^9^ Subsequent studies have demonstrated that CRCDS patients exhibit a unique ventricular repolarization response following either spontaneous or electrically induced brief tachycardia and subsequent pause (LBLP protocol without the extra-stimulus). This response, detectable as changes in the QT interval and T-wave amplitude referred to as ‘T-wave ballooning’, is absent in healthy individuals or in patients with CPVT1.^13^ One of our six putative CRCDS patients in the IVF cohort underwent EP study; although no VAs were induced, the characteristic T-wave ballooning response was indeed present, suggesting that the LBLP protocol may assist in detecting CRCDS. However, further studies are needed to determine whether the protocol requires temporary discontinuation of β-blocker/flecainide therapy to unmask this response, as current evidence is mixed^13,38^—a factor that may discourage patient participation.

Among our 6 patients, all RyR2 variants were initially classified as VUS. In one case, the variant (p.R4608Q) was identified as *de novo*, and functional studies confirmed a LOF effect in RyR2,^29^ allowing for confident reclassification of the variant as pathogenic. In one additional case, the variant was upgraded to pathogenic based on supporting functional data (p.G4935R).^30^ In the remaining four cases, the diagnosis of CRCDS was based on the clinical phenotype of exertion-related SCA, combined with the identification of an ultra-rare nonsynonymous variant located within a functionally important region of RyR2, supporting a likely pathogenic classification rather than VUS. However, we emphasize that the presence of an ultra-rare RyR2 variant in a patient with a normal treadmill stress-test should not be interpreted as sufficient for a CRCDS diagnosis; a definitive diagnosis still requires either a positive CRCDS electrophysiology study or *in vitro* functional demonstration of LOF effect of the RyR2 variant.

The human *RYR2* gene, localized on chromosome 1q43, encompasses 105 exons coding for the 4967 amino acid RyR2 monomer that forms homo-tetrameric complexes embedded in the SR membrane. Each monomer consists of a large cytoplasmatic N-terminal domain (representing ∼90% of entire protein) with a characteristic mushroom-like shape and a C-terminal region, including the transmembrane pore-forming segments (S1–S6) and the luminal domain exposed to the SR (S5–S6 loop) containing the selectivity filter.^39^ The RyR2 C-terminal (∼500 residues) plays an important role in channel function, regulating ATP, luminal Ca^2+^ binding, but also is critical for maintaining its closed state during diastole, preventing Ca^2+^ leak and afterdepolarization formation that may initiate arrhythmia.^40^ To ensure physiological function, dynamic regulatory mechanisms, ranging from post-translational modifications to ligand-based mechanisms, regulate open probability and gating properties of this Ca^2+^ channel.

Prior studies have demonstrated that pathogenic variants in RyR2 associated with IVF and congenital arrhythmia syndromes, including CPVT1 and CRCDS, tend to cluster in specific loci (clusters I to IV).^41,42^ While no apparent correlation has been found between variant location or whether the clinical presentation aligns with ‘typical’ or ‘atypical’ CPVT1 phenotype, variants located in the N-terminus appear to be associated with a prolonged QT interval.^43^ Interestingly, variants located in the C-terminal domain manifest a significantly higher VA burden and increased risk of β-blocker failure when compared to N-terminal variants.^32,44^ In our study, the majority of CRCDS-predisposing *RYR2* variants identified in our IVF patients or SUDY victims (∼80% overall) localized to the clusters III and IV, corresponding to the central and C-terminal domains, respectively.

Genetic arrhythmia syndromes associated with RyR2-pathogenic variants may result from several molecular mechanisms, including disrupted RyR2 inter- and intra-subunit interactions, interactions with accessory proteins, altered cytosolic/luminal Ca^2+^ activation, or abnormal Ca^2+^ conductance.^39^ These mechanisms appear to be relevant for both CPVT1 and CRCDS. However, cellular and animal functional studies suggest a distinct arrhythmogenic mechanism in CRCDS, including but not limited to, Ca^2+^ homeostasis dysregulation. Unlike RyR2-GOF variants which promote increased spontaneous SR Ca^2+^ release, RyR2-LOF variants significantly reduce or abolish SR Ca^2+^ leak and exhibit lower risk for stress-induced VA following caffeine or isoproterenol treatment, compared with WT RyR2 channels.^9,32,45,46^ Collectively, these findings indicate that the arrhythmogenic mechanism in CRCDS operates independently of the abnormal RyR2 opening or improper channel closure characteristic of CPVT1.^37^

In CRCDS, the impaired RyR2 activity leads to reduced SR Ca^2+^ release and subsequential accumulation of Ca^2+^ within the SR, which may alter the threshold for store overload-induced Ca^2+^ release, a proposed key arrhythmogenic mechanism.^2,32^ To date, studies have shown that RyR2-LOF variants prompt major electrophysiological remodelling, possibly reflecting compensatory molecular adaptations that may be central to CRCDS arrhythmogenesis.^9^ These include altered sarcolemmal Ca^2+^ inflow/outflow through the L-type Ca^2+^ channel and NCX pump respectively. Notably, the electrical remodelling extends beyond Ca^2+^ handling, also affecting K^+^ and Na^2+^ currents, such as the transient K^+^ outward current (I_to_) and the sodium current (I_Na_).^9^

### Limitations

This study has several limitations. First, our cohort was derived from a tertiary care genetic heart rhythm specialty centre, which may not be representative of patients seen in primary or secondary care settings. Second, for the majority of cases exercise stress testing was performed while patients were on anti-arrhythmic medications. Although testing off medication would provide more accurate diagnostic information, this was not feasible or safe as many patients had experienced prior SCA and required ongoing treatment. Third, not all RyR2 variants have been characterized functionally, reflecting the current challenges in functionally assessing RyR2 variants due to the complexity and limitations of available assays. We acknowledge that this represents a significant limitation in determining the pathogenic contribution of ultra-rare RyR2 variants. To address this gap, a systematic experimental assessment of RyR2 variants will be essential for refining variant classification and informing clinical diagnostic and therapeutic decisions.

## Conclusions

Our findings highlight CRCDS as a distinct and underrecognized clinical entity within the spectrum of exertion-related SCA. Despite normal exercise stress test results, patients with CRCDS remain at significant risk for life-threatening arrhythmias. The identification of CRCDS in up to 9% of patients with exertional SCA who had been referred to our institution as IVF and at least 7% in SUDY cases with an exertion-related SCD emphasizes the need for heightened clinical suspicion for CRCDS, especially in the presence of RyR2 VUS in the setting of exertion-related triggered events. Early recognition of this phenotype is critical for appropriate management and prevention of SCA.

## Supplementary Material

euaf303_Supplementary_Data

## Data Availability

The data underlying this study consist of clinical and genetic information obtained from patients diagnosed with IVF and decedents classified as SUDY; due to ethical restrictions and the sensitive nature of the patient data, these datasets are not publicly available. De-identified data supporting the findings of this study may be made available to qualified researchers upon reasonable request to the corresponding author.
