# Supplementary material for: Calcium release channel deficiency syndrome in patients diagnosed with idiopathic ventricular fibrillation and decedents classified as sudden unexplained death in the young
Source: Europace. 2026 Mar 5;28(2):euaf303. doi: 10.1093/europace/euaf303 (PMC12962230; doi:10.1093/europace/euaf303)
Supplement: euaf303_Supplementary_Data [file euaf303_supplementary_data.docx]

**SUPPLEMENTAL MATERIAL**


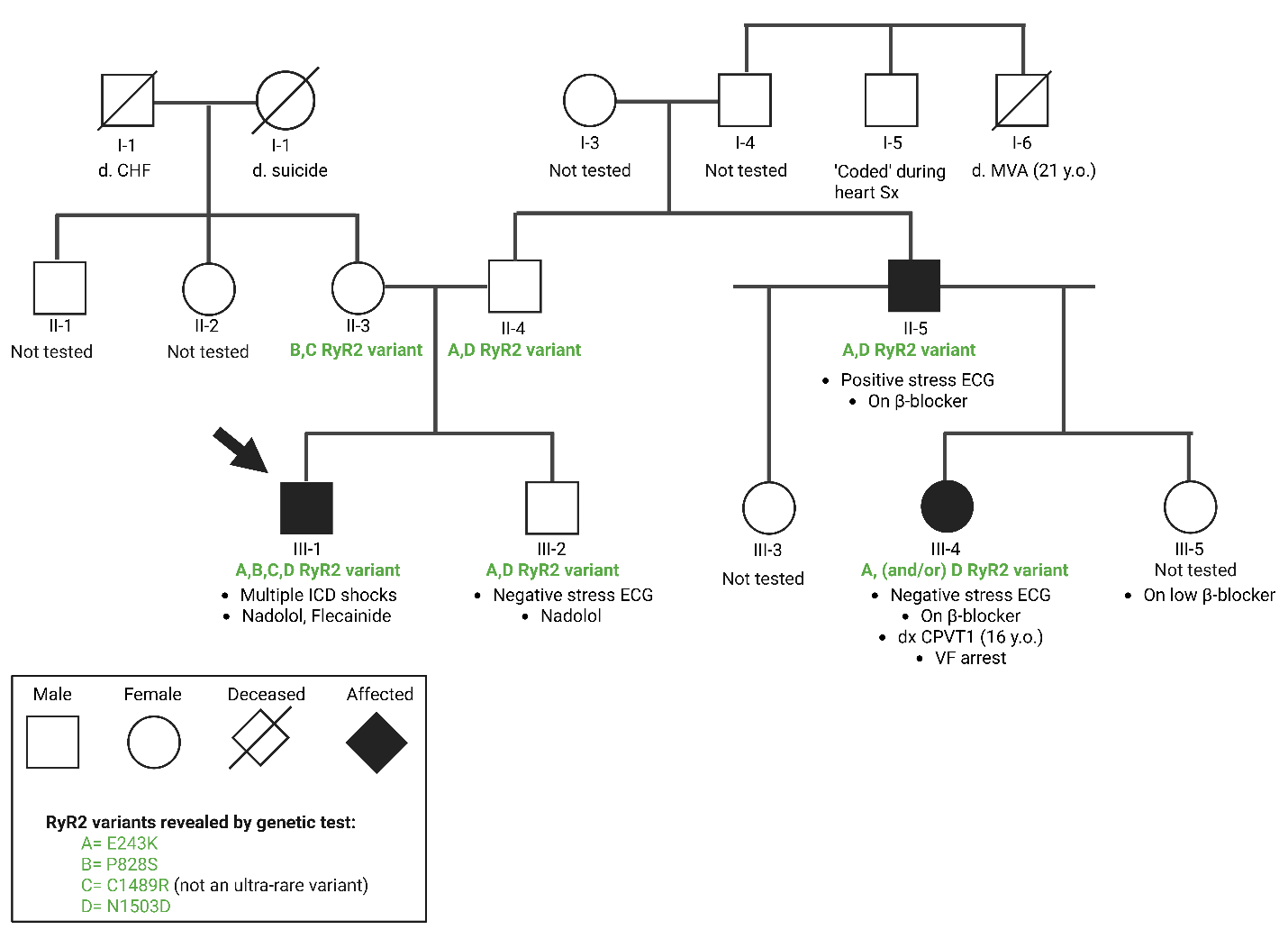
**Supplemental Figure 1: Genetic pedigree of the first CRCDS case identified in the IVF cohort**

Abbreviations: CPVT1, catecholaminergic polymorphic ventricular tachycardia type 1; d, died; ECG, electrocardiogram; ICD, implantable cardioverter-defibrillator; MVA, malignant ventricular arrhythmia; sx, surgery; VF, ventricular fibrillation.

**Supplemental Table 1: Summary of *RYR2* variants identified in SUDY victims**

|  | **Sex** | **Age (years)** | **Race** | **SCD Circumstance** | **Personal/Family History of Cardiac Events** | **Nucleotide Change** | **Amino Acid Chage** | **Function** | **Inheritance** |
| --- | --- | --- | --- | --- | --- | --- | --- | --- | --- |
| 1 | F | 10 | W | Exertion | FH: SCD | Homozygous 344,085 bp duplication involving ~26,000 bp of intergenic sequence, 5’UTR/promoter and exons 1-4 | Homozygous Exon 1-4 Duplication | LOF | Familial Recessive |
| 2 | M | 17 | W | Exertion | FH: SCD | c.37T>C | p.F13L | GOF | Maternal |
| 3 | M | 21 | A | Nonspecific |  | c.338T>C | p.L113P | NA | De novo |
| 4 | F | 22 | A | Nonspecific | PH: Syncope,  FH: SCD | c.341T>C | p.L114P | NA | NA |
| 5 | M | 2 | W | Exertion |  | c.719A>G, c.12472A>C | p.H240R, p.T4158P, | NA, GOF | NA |
| 6 | M | 16 | W | Exertion |  | c.854G>A | p.S285N | NA | NA |
| 7 | F | 35 | W | Nonspecific | FH: SCD | c.1198G>C | p.D400H | NA | NA |
| 8 | M | 9 | H | Nonspecific | PH: Syncope | c.1220G>T | p.R407I | NA | NA |
| 9 | F | 16 | W | Drowning | PH: Syncope,   FH: SCD | c.1240C>T | p.R414C | NA | Paternal |
| 10 | F | 13 | W | Drowning | FH: SCD | c.1255A>T | p.I419F | NA | Paternal |
| 11 | M | 17 | W | Exertion | PH: Syncope | c.1258 C>T | p.R420W | GOF | Maternal |
| 12 | F | 22 | W | Nonspecific | FH: SCD | c.1258 C>T | p.R420W | GOF | NA |
| 13 | M | 25 | W | Exertion |  | c.3788 C>A | p.S1263X | NA | NA |
| 14 | M | 16 | W | Nonspecific |  | c.6683G>T | p.G2228V | NA | NA |
| 15 | M | 5 | W | Exertion |  | c.6739 C>T | p.S2246L | GOF | NA |
| 16 | M | 4 | B | Exertion |  | c.6739 C>T | p.S2246L | GOF | NA |
| 17 | M | 14 | W | Drowning |  | c.6962T>C | p.V2321A | NA | Maternal |
| 18 | F | 25 | W | Exertion | FH: SCD | c.7175A>G | p.Y2392C | NA | NA |
| 19 | F | 8 | W | Drowning | PH: SCA | c.7202G>A | p.R2401H | NA | De novo |
| 20 | M | 9 | W | Drowning |  | c.7423 G>T | p.V2475F | GOF | NA |
| 21 | F | 17 | W | Nonspecific |  | c.7528A>G | p.T2510A | NA | NA |
| 22 | F | 9 | W | Exertion | PH: Syncope | c.11773 C>G | p.Q3925E | LOF | NA |
| 23 | F | 15 | W | Exertion |  | c.11636T>C | p.S3959L | NA | NA |
| 24 | M | 16 | W | Exertion |  | c.11919T>G | p.D3973E | NA | Maternal |
| 25 | M | 18 | W | Nonspecific | FH: SCD | c.12290A>G | p.N4097S | NA | Paternal |
| 26 | M | 14 | W | Sleep | FH: SCD | c.12436 G>A | p.E4146K | LOF | NA |
| 27 | F | 34 | B | Nonspecific | FH: SCD | c.13610 C>T | p.R4497C | GOF | NA |
| 28 | F | 28 | W | Sleep |  | c.13655A>G | p.H4552R | GOF | NA |
| 29 | F | 5 | W | Exertion | PH: SVT | c.13933 T>A | p.W4645R | LOF | De novo |
| 30 | M | 14 | W | Exertion |  | c.14288A>G | p.N4763S | NA | NA |
| 31 | F | 8 | W | Exertion | PH: Seizure,   FH: Syncope | c.14803 G>A | p.G4936R | LOF | De novo |

Abbreviations: A, Asian; B, Black; F, Female; FH, Family History; GOF, Gain-of-Function; LOF, Loss-of-Function; M, Male; NA, Not Available; PH, Personal History; SCA, Sudden Cardiac Arrest; SCD, Sudden Cardiac Death; SVT, Supraventricular Tachycardia; W, White.
